# DNA storage—from natural biology to synthetic biology

**DOI:** 10.1016/j.csbj.2023.01.045

**Published:** 2023-02-02

**Authors:** Elena Bencurova, Aman Akash, Renwick C.J. Dobson, Thomas Dandekar

**Affiliations:** a Department of Bioinformatics, University of Würzburg, Würzburg, Germany; b Biomolecular Interaction Centre, University of Canterbury, Christchurch, New Zealand; c Department of Biochemistry and Pharmacology, University of Melbourne, Melbourne, Australia; d Structural and Computational Biology, European Molecular Biology Laboratory, Heidelberg, Germany

**Keywords:** DNA, RNA, Data storage, Natural processing, Synthetic biology

## Abstract

Natural DNA storage allows cellular differentiation, evolution, the growth of our children and controls all our ecosystems. Here, we discuss the fundamental aspects of DNA storage and recent advances in this field, with special emphasis on natural processes and solutions that can be exploited. We point out new ways of efficient DNA and nucleotide storage that are inspired by nature. Within a few years DNA-based information storage may become an attractive and natural complementation to current electronic data storage systems. We discuss rapid and directed access (e.g. DNA elements such as promotors, enhancers), regulatory signals and modulation (e.g. lncRNA) as well as integrated high-density storage and processing modules (e.g. chromosomal territories). There is pragmatic DNA storage for use in biotechnology and human genetics. We examine DNA storage as an approach for synthetic biology (e.g. light-controlled nucleotide processing enzymes). The natural polymers of DNA and RNA offer much for direct storage operations (read-in, read-out, access control). The inbuilt parallelism (many molecules at many places working at the same time) is important for fast processing of information. Using biology concepts from chromosomal storage, nucleic acid processing as well as polymer material sciences such as electronical effects in enzymes, graphene, nanocellulose up to DNA macramé , DNA wires and DNA-based aptamer field effect transistors will open up new applications gradually replacing classical information storage methods in ever more areas over time (decades).

## Graphical Abstract

**Figure:**
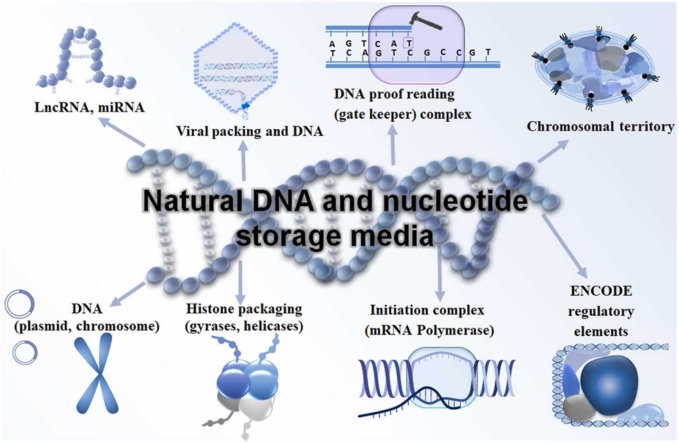

## 1 Introduction

Our world is increasingly digital and the amount of data created is enormous. Such data can be stored in conventional storage devices; however, with the enormous increase in data, but requires large volumes of physical space and energy. To overcome this, several alternatives are currently being developed or used for specific applications, such as DNA, which has opened up exciting possibilities for high-density storage [25]. However, there is still a long way to go before DNA storage is as simple as using USB sticks, SSD discs and other available electronic storage devices. How can we make it possible? We believe we should learn a little lesson from nature: DNA storage works well in chromosomes and even in individual genes. Here, information stored in DNA is rapidly and efficiently transported via RNA to the most distant parts of the cell, while RNAs regulate, transform and edit the existing information. Interestingly, the approach of data storage is very well achieved in the brain—our thinking is based on the neurons, which allows a very high level of calculation and fascinating RNA storage, including microRNA (miRNA) and long non-coding RNA (lncRNA) for the reading, processing and delivering of information.

Despite the recent advances in synthetic biology, for example the appearance of the “DNA fountain” [24] and the “DNA harddrive” [15] as devices, there are major obstacles that prevent the application of DNA data storage for routine use. In this review, we present biological insights on current storage systems to learn from nature and promote DNA and other nucleotides as an attractive alternative to current storage systems, from the current electronics up to molecular technology. However, without the next or over-next level of technology including the next industrial revolution, progress will be difficult, or even impossible to attain.

## 2 Perspective: Insight from biology

DNA is the pivotal molecule of every living organism because it contains the instructions for cell development, reproduction and survival. DNA contains millions or billions of base pairs, so it has to be transcribed into messenger RNA (mRNA) before the specific information for protein synthesis can be translated into proteins at the ribosome. Rapid access to specific segments of the DNA molecule that encode the necessary genes for protein transcription/translation is crucial for adaptation strategies and rapid development during stress, starvation or harmful conditions, therefore the regulation and protein synthesis occurs through an intermediate molecule, RNA. The cell uses a very efficient RNA and DNA storage concept that provides inspiration for artificial data storage.

### 2.1 Cellular DNA storage: Operated at micro and nano-level

**Fig. 1:**
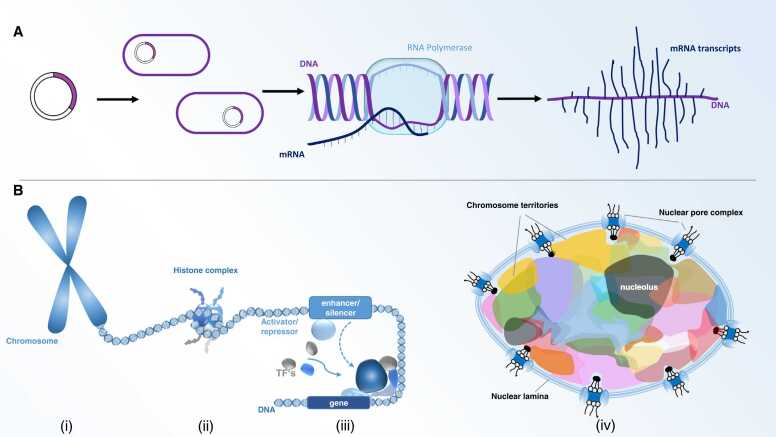
A: Streamlined DNA storage in bacteria allows fast read-out, processing and fast read-out. Left: Plasmid rings as the smallest unit. Larger structures: DNA chromosome with RNA polymerase. Right: mRNA is direct transcribed and translated (“Christmas tree” structures). B: Extravagant, much larger DNA storage in eukaryotes. This spread-out DNA storage contains 1000-fold more storage space, allows for sophisticated access and different levels of information release, incorporation and modification (methylation patterns, miRNAs, RNA editing, mRNA, histones etc. see text). Left: Chromosome, next DNA with histones forms nucleosomes. Middle: Activator/repressor and enhancer/silencer and, towards the middle, transcription factor complexes. Right: chromosomal territories.

Unlike higher organisms, bacteria lack a nucleus and their DNA is stored in the nucleoid, the concentrated single circular molecule of DNA attached to the cell membrane directly in the cytoplasm. In addition, many bacteria also contain further DNA molecules in form of plasmids, which provide evolutionary benefits, such as genes for antibiotic resistance, toxins production, and in some cases facilitate bacterial replication [54]. Bacteria (Fig. 1, up) are one of the fastest reproducing organisms, with a doubling time from 4 min to several days. Such fast replication can be challenging and is often limited by the need for proofreading and error corrections, which in turn limits their DNA length to a few million nucleotides and works to prevent high error occurrence. Such errors are responsible for the genetic mutations that influence the overall fitness of the cell and impact cell viability and functionality. This limit can be so strict that important information such as resistance to antibiotics can be lost if selection pressure is missing. Consequently, bacteria rapidly lose antibiotic resistance plasmids if conditions are no longer selected for their presence.

With the appearance of the eukaryotic cell about 2 billion years ago [72], a new generation of DNA storage was introduced (Fig. 1, down). The DNA molecules were no longer housed in the cytoplasm and were separated in the nucleus; thus, the storage compartment is distinguished from direct transcription by controlling the nuclear pore storage apparatus. In addition, improved storage capabilities and better control have enabled 1000 times larger storage capacities compared to the prokaryotic cells. This means that storage is no longer critical for the evolution of complexity and thus complexity no longer correlates with DNA length in eukaryotes (DNA content and gene paradox, reviewed in [16]).

#### 2.1.1 Access to DNA storage at the micro-level

In a eukaryotic cell, DNA is stored in the nucleus, which is separated by a double membrane from the site of protein production and other cell processes. In addition, several other mechanisms ensure fast, efficient and easy-to-handle storage and processing of information stored in DNA:

*Chromosomes and chromosomal territories:* Eukaryotic DNA is not a single molecule, as seen in bacteria, but is instead crowded into individual packages called chromosomes that are easily visible under the microscope. From the point of view of synthetic biology, chromosomes are wonders in enabling rapid access and efficient storage. Moreover, together with the ribosome and the cytoplasm, are also capable of the production of proteins at critical moments. At the molecular level, the tasks of accessing DNA storage in a controlled (regulated) manner is undertaken with the help of transcription factors (TFs) and the packaging of important information into individual genes provides better access. And here comes the first enhancement, how to improve digital data storage inspired by biology.

Typical problems of storage are RAM (rapid access), precision, recall and retrieval. In electronic storage, these problems have been solved, but this is not the case for artificial DNA storage. On the other hand, natural DNA storage in chromosomes, however, works surprisingly efficiently [63] and even the integration of new DNA and mobile elements is surprisingly efficient (Vial and Hommais, 2019).

To enable efficient readout of genomic information, chromosome orientation and displacement correlate with gene expression, which depends on cell geometry and predictably influences gene expression patterns [65]. Ellipsoid packing as well as mechano-sensitivity of chromosomes allow their optimal orientation in the nucleus. Transcription inhibition experiments show that global changes in transcription affect chromosomal position and orientation. Biological DNA storage provides efficient readout and genome regulation, actively controlled in hotspots where chromosomes intermingle (called chromosomal territories) [63]. The edges of chromosome territories allow the formation of rare long-range inter-chromosomal interactions with chromosome translocations based on physical proximity [60]. Thus, in eukaryotic organisms, specific information about the genome can be accessed by micro-arranging the DNA strands, which is another attractive property that can be exploited by synthetic biology for synthetic DNA storage. The architecture of such a data storage scheme includes nucleosomes as the smallest chromatin building blocks, the next level is Mbp chromatin domains and finally topologically associating domains (TADs) which are integrated into chromosome territories [26]. To stimulate readout, enhancers loop back to interact with their target genes in topologically associating domains, for a common transcription set of genes and chromatin domains allow dynamic motion in vivo. Furthermore, interchromosomal cell type-specific networks of chromosome territories that change with cell cycle and differentiation could be generated by mining the image graph. Interchromosomal cell type-specific networks are proposed to correlate with the global level of genome regulation.

*Mobile storage in plasmids and chromosomal interaction:* As noted above, plasmids are circular DNA molecules for storing additional biological information that is beneficial for environmental adaptation and/or has evolutionary benefits. Plasmids are typically found in bacteria, but they can also rarely occur in eukaryotes, and even they have important functions in molecular biology (e.g., transformation of plant cells using *Agrobacterium tumefaciens*). Interestingly, there is a crosstalk between the plasmids and chromosomes. Chromosomal regulators can control the expression of plasmid functions and vice versa, several plasmid-encoded regulators were identified that regulate chromosomal genes [64] and thus both are mutually responsible for the long-term maintenance as well as the daily readout of both types of DNA storage.

#### 2.1.2 DNA storage access operated at the nano-level

*DNA polymerases*: DNA polymerases are ubiquitous that are involved in the synthesis of nucleotide polymers. Nevertheless, there is a growing potential for their use in synthetic biology, given their high biological richness and diversity on which applications can be developed [19]. In particular, DNA polymerases are involved in the regulation of DNA storage [68] as they either synthesize mRNA for repair enzymes or help directly in DNA repair besides involvement in replication which has higher rates of intrinsic error. In a live organism, there is a large number of specialized polymerases that protect against different errors (such as broken DNA ends, nucleotide incorporation, radiation damage etc.). Together they increase the stability of the genome and thus of the stored information, which is why such enzymes can also be used in artificial DNA storage. The traditional DNA polymerases used for the replication can be engineered in numerous ways for biotechnology, exploiting their efficient and accurate amplification of DNA templates for desired functions using mutagenesis or through the creation of protein chimaeras. New techniques help, for instance, selection in water-in-oil emulsions instead of phage display selection [46]. Engineered DNA polymerases withstand extreme conditions or the presence of inhibitors, as well as polymerases with the ability to copy modified DNA templates [19]. The engineering of mammalian polymerases is attractive for optimizing nucleotide information storage as recently detailed structure information became available [34]. A well-known and constantly improving application is transcriptome reading and DNA storage. For transcriptome reading, one can use PacBio full-length cDNA and DNA sequencing [73]. Moreover, optical mapping may become a future rapid alternative strategy for efficient DNA storage [71]. Template-free polymerases may be flexible polymerases for future storage applications [20], [7].

#### 2.1.3 Possibilities of information processing through RNA

The concept of artificial DNA data storage has been known for a long time, but also ribonucleic acid (RNA) could also be used for data storage. However, RNA is not just a simple molecule with a uniform function. There are many types of RNA that vary in length from a few nucleotides to several thousand, with many roles in gene expression regulation and their processing, and protein production and some of them can even defend against RNA viruses. In a cell, mRNA carries a blueprint of the stored information for swift processing, keeping the original genetic information preserved. RNA storage can be a useful alternative for biotechnology applications, efficiently produced by different RNA polymerases [7]. For instance, RNA cantilevers can be applied as biosensors to detect RNA and DNA for stored data access [32].

*LncRNA*: Larger sections of the DNA storage can be blocked or released by large non-coding RNAs [59]. There are at least 16,000 lncRNAs known, but their exact amount may exceed 100,000. Although not well characterised, they have multiple and often vital functions for the organism. They allow gene silencing, chromatin regulation, and X chromosome inactivation including shaping the 3D architecture of the inactivated DNA. In general, the 3D structure of chromosomes also regulates the transcription of chromosomal DNA storage, which directs transcription factors to appropriate reading sites [39].

In addition to transcriptional gene regulation by lncRNA, there is also posttranscriptional regulation, such that non-coding RNA activated by DNA damage (NORAD) acts as a decoy of the Pumilio RNA-binding proteins, which mediates post-transcriptional repression of DNA replication and repair factors. By binding to a protein, nuclear NORAD is involved in the maintenance of topoisomerase 1-mediated biological DNA storage [59]. Moreover, transcription of the lncRNA locus exerts regulatory functions using transposable elements (TEs), important mobile genomic elements that are widely represented in the eukaryotic genome [4]. One example is the primate-specific lncRNA *XACT* that protects the active X chromosome from being silenced by *XIST* lncRNA whose sequence again contains elements derived from a TE. *XIST* lncRNA silencing is reviewed by [11], however, it plays the role of a cancer marker and a new therapeutic target [55]. Furthermore, lncRNA location signals allow the specific release of information which are stored here [10]. This capability can be used in synthetic biology expression constructs (simplest application: use the position control element to direct the location of RNA expression in ones eukaryotic expression system of choice) or to direct the location of storage units within a synthetic biology organism for nucleic acid storage.

*Piwi-interacting RNAs* (piRNAs) are also interesting for future applications, as they keep the TEs under control in eukaryotic cells directing DNA methylation, and thus releasing the stored information in the DNA [74].

*rRNA, catalytic RNA*: These molecules are used both in biology and synthetic biology to translate the stored information (DNA or RNA) into biological activity (enzymes) through the formation of the peptide bond by catalytic rRNA [37]. Modifications such as the artificial ribosome or catalytic RNA for ribozyme activities [13] are highly interesting applications for engineered RNA. Moreover, miRNAs modulate mRNA stability. Thus, information storage in nucleotides can be modulated by oligonucleotides in various ways, for instance using therapeutic oligonucleotides to eliminate specific mRNAs [62].

Design protocols for RNA ligase ribozymes have been established [48] and offer a blueprint for further ribozyme design activities. There is even programmable formation of catalytic RNA triangles and squares by assembling modular RNA enzymes [49]. Workflows use inverse RNA folding to design and test ribozymes that include pseudoknots (Kayedkhordeh et al., 2021).

**Fig. 2:**
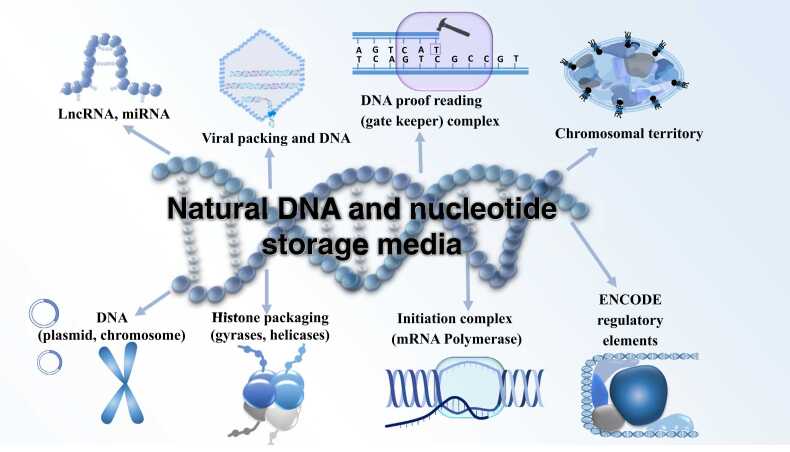
Natural DNA and nucleotide storage media. Lnc RNA, miRNA; viral packing and DNA; DNA proofreading (gate keeper) complex; DNA (plasmid, chromosome); Histone packing (gyrases, helicases); initiation complex (mRNA polymerase); ENCODE regulatory elements; chromosomal territories.

*Use RNA storage molecules as flexible detectors*: The same blueprint is used everywhere, but methylation, transcription factors, and chromosomal territories (see above and Fig. 2) ensure that each cell type gets its correct version transcribed into RNA, ignoring other genetic information. At the RNA level, RNA cantilevers enable the detection of genes and mutations, such as BRAF mutations in melanoma cells [35], or antimicrobial resistance genes in bacteria [36].

### 2.2 RNA binding proteins and transcription factors

RNA binding proteins (RBPs) modulate the action of the RNAs on DNA storage, particularly useful to achieve transcriptional control of the DNA/RNA storage through diverse RBP-chromatin interactions [21]. In contrast, transcription factors though central to the biological mechanism of DNA reading, are currently too complex to regulate, requiring assembly into large protein complexes, challenging to use in DNA storage operations. But that may soon change, e.g., the structure of the human mediator-bound transcription preinitiation complex has become available in the past year [1] and also the organization and regulation of chromatin are becoming clearer, for instance in plants [42] and regarding individual cells [12].

### 2.3 Genome editing

The role of RNA in living organisms is versatile and closely connected with the information stored in DNA, but at the same time, it also can modulate it. Using RNA, it is even possible to directly manipulate any gene or genome in a desired way. This means editing the DNA storage in a specific location within the genome is possible with current technologies. There are sets of specific engineered nucleases, that contain DNA-binding domains to target specific sequences as well as non-specific nucleases that can specifically cleave DNA and precisely modulate genetic information. This process is called genome editing.

Procedures for gene replacement in baker’s yeast, *Saccharomyces cerevisiae*, have been available for several decades. Using the yeast gene conversion mechanism to replace one gene with an alternative, engineered or modified version and then apply it to the higher organisms, particularly mice as model organisms. Without this process, it would be necessary to apply tedious procedures and long screening until the desired modification is obtained. Since around 2003, zinc finger nucleases (ZFN) have been known to be suitable for genome engineering [14] by the usage of ZFN-induced double-strand breaks that are subject to cellular DNA repair processes. This approach can be used for targeted mutagenesis and targeted gene replacement at remarkably high frequencies [14]. Other alternatives for genome editing are Transcription-Activator Like Effector Nucleases (TALEN) and Clustered Regularly Interspaced Short Palindromic Repeats-CRISPR-Associated 9 (CRISPR-Cas9) [30]. The latter is an effective, fast, simple and cheap technique for knocking out genes and editing the genome by focusing on double-strand breaks in the organism and the selected cell type. Using paired nicks prevents off-target effects of the CRISPR-Cas system [30]. Moreover, it was shown that the CRISPR array can be used to effectively store text data in live cells (Yim, McBee et al., 2021), where data are stable over 50 generations [56].

## 3 Natural processing power: all works in parallel

*Using DNA storage molecules in parallel:* There is another advantage of natural storage—it has built-in parallelism. Here, instead of using just one molecule of DNA, each cell contains a full copy of building plans (chromosomes) for development, replication, regulation, and survival, which means there are billions of genes existing in parallel. Only with such a distributed storage is cellular differentiation possible. Therefore, the now topical cellular automata [66] for computer simulations and in particular the modern agent-based simulations for cellular processes [58] have long been already implemented by our cells just to make the execution of our bodybuilding plan possible.

However, the use of DNA parallelism has long been known for mathematical calculations and synthetic biology. DNA provides a huge amount of computational potential that can be used for complex arithmetic and logic calculations that can be calculated only with the help of supercomputers (so-called natural computing). A well-known first example was solving the travelling salesman problem by ligating DNA sticks of different lengths together and considering many DNAs in parallel [2]. Other applications have also been solved using DNA computing since for instance another distribution problem, the Chinese Postman problem [70]. However, since electronics and modern silicon chips already work as very fast processors, DNA computing is not yet competitive in this respect. However, in other aspects, especially in high-density DNA storage (Exabyte per gram, [25]) and long-term storage DNA is a superior storage medium [29]. Moreover, promising new processing and programming applications use programmable connectivity of DNA to achieve programmable material, such as nanoparticles interacting with DNA bonds [53], or to design hierarchical assembly pathways of proteins with DNA to manufacture protein-DNA material with a high level of complexity [33]. These nano-structuring capabilities of DNA prove that processing and programming with the help of DNA have enormous potential and can solve problems that are inaccessible to other fields such as chemistry and electronics [23]. Moreover, chemical modification opens a new horizon of opportunities, which other branches can further optimize using nucleic acids.

## 4 How does technical DNA storage look like?

**Fig. 3:**
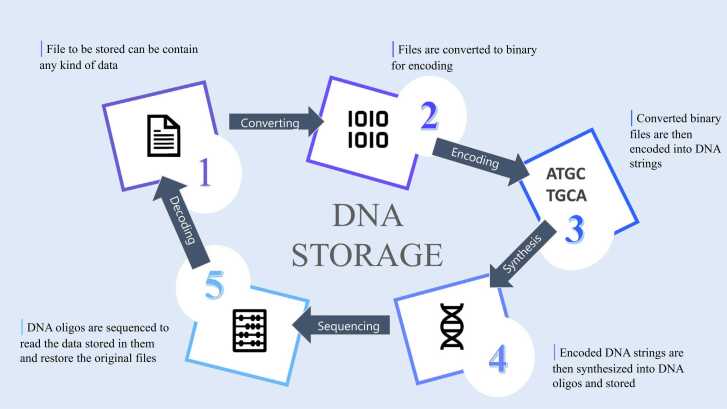
Simple technical DNA storage. You start from (1) file to be stored (text, image, sound); (2) next there is the conversion into code and a suitable binary file (3). You then synthesize the DNA and have the encoded DNA strings (4); For decoding the DNA oligos are sequenced (5) → From this information, the original data file is regenerated (text, image, sound).

**Fig. 4:**
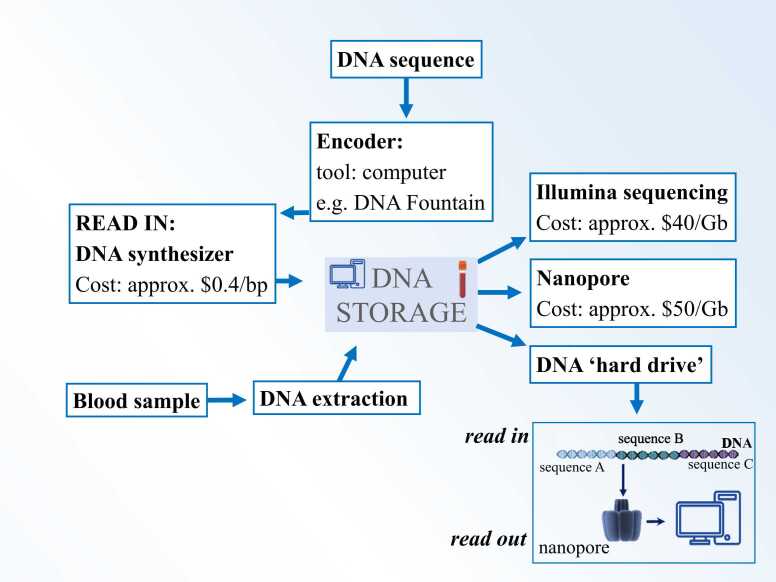
State-of-the-art DNA storage: Top: The DNA sequence of choice should be encoded with error correcting codes in a PC. Then (follow the work flow, blue arrows) a synthesizer allows to synthesize the DNA sequence (left). Next, the obtained DNA is stored in a DNA storage device (Icon in the middle). Bottom, left: natural DNA sequences from patients such as blood samples can also be easily stored in the DNA storage device. Right: Storage read-out (follow arrows): By Illumina or Nanopore sequencing. Cartoon at bottom right: Modern lab methods include the DNA hard drive. DNA Sequences to be stored are anchored to a stable and large DNA backbone molecule; fast read-out is done by latest nanopore sequencing.

Currently, we are very close to realizing the industrial application of DNA-based storage. In a pioneering work, Church published the high coding potential of DNA [17] and a year later Goldman et al. [28] demonstrated that text, sound, and images can be efficiently stored in DNA. Moreover, the potential storage has been calculated to be 455 Exabyte/g of DNA [17], 1 Exabyte are 10^18^ bytes (Exstance, 2016) and taken together, this laid the basis for further impressive steps in constantly improving DNA storage. An exemplary process flow of storing digital data in DNA is shown in Fig. 3. In particular, Erlich and Zielinski [24] showed that the encoding scheme “DNA Fountain” enables a robust and efficient storage architecture and further technical improvements allowed Church’s group to demonstrate photon-directed multiplexed enzymatic DNA synthesis for molecular digital data storage [43]. How does the state-of-the-art DNA storage look like? This is illustrated in Fig. 4: We need to use a PC to design error correcting DNA sequences with check-sums and test bits. Next a synthesizer allows to synthesize the DNA sequence with cheap costs of only $0.4/bp. The new DNA is then stored in the DNA storage. Currently a range of solutions for the actual storage include sequencing slides, test tubes or protective polymers around the DNA such as nanocellulose. Importantly, in modern clinics (university clinics, human genetics laboratories etc.) there is another direct access route: the natural DNA sequence from the patient is put into the DNA storage repository taking blood.

There is also a range of possibilities for getting the read-out from the storage: Illumina or Nanopore sequencing (typical costs 40–50 dollars per Gigabyte) and there are more fancy methods at least tested in the lab such as the DNA hard drive (cartoon in Fig. 4, building something like an artificial chromosome using a DNA backbone), so that we expect the prices to fall even more in the near future.

Moreover, Table 1 gives an overview on current methods in place for making such a DNA storage work, such as error correction methods specialized for DNA storage, PCR-based random access techniques, different synthesis/sequencing technologies (array-based synthesis, enzymatic synthesis, short read vs. long read nanopore sequencing).

**Table 1:** Learning from Biology: Current methods for DNA storage in place.

**Methods**	**Overview**	**Reference**
Synthesis Techniques	Enzymes like TdT can be used to synthesize synthetic DNA having good length for DNA storage while giving more control over the synthesis and does not suffer from environmental disadvantages which arise from chemical synthesis techniques. Array-based synthesis has also improved such that they enable parallelized light-gated synthesis favourable for DNA storage.	Lee et al., [44] Lee et al., [43] Antkowiak et al.,[6]
Sequencing Techniques	Short-read sequencing still dominates sequencing practices but long-read sequencing like nanopore which utilizes natural transmembrane proteins could prove more useful in DNA data storage due to their ability to produce longer reads. The downside of losing accuracy can be balanced using error-correcting schemes.	Zhang et al.,[73] Lopez et al.,[45]
PCR-based techniques	PCR in DNA storage is typically used for amplification of the stored data for error-free data retrieval, but it can also be used for random access and re-writability of the stored data.	Tabatabaei Yazdi et al.,[61]
Error Correction methods	Data storage in DNA is highly prone to errors which arise from synthesis, sequencing or just random loss. Error-correcting schemes can be utilized to overcome these errors and the successful retrieval of stored data. Famous error-correcting codes like Reed-Solomon are widely used but specific error-correcting codes tailored for DNA storage like HEDGES diminish error rates.	Blawat et al.,[9] Press et al.,[50]
*In-vivo* storage	Crispr-CAS9 system can be used for gene editing and storage of information directly in the cells. This allows increased stability and replication of the stored data. Parallelism and multiplexing can also be achieved for in-vivo storage which makes it promising. The basic disadvantage of mutation can easily be solved by the incorporation of error-correcting schemes.	Shipman et al.,[57] Yim[69] Akhmetov et al.,[3]

However, a couple of significant problems remain: error correction and certainly biological ambiguity such as mutations and sequencing errors, that are not present in other storage devices. Sequencing errors can be avoided by two or more rounds of sequencing, but despite the low cost of sequencing compared, this remains an additional cost that may prevent the use of DNA as a large-scale storage medium (synthesis: Megabytes; read-out, sequencing: Gigabytes and beyond). On the other hand, there are many applications of pragmatic DNA storage for biotechnological use (e.g., DNA used for nano-structuring materials) and for preserving DNA as important genetic material on coverslips and advanced storage devices including deep freezing (−70 °C or −177 °C in liquid nitrogen), which are very useful for long-term preservation of human genetics samples, as this guarantees rapid access and sequencing. Moreover, biological materials can be preserved alive (e.g., stem cells from umbilical cord blood stored at birth). After considering all the facts, Fig. 2 shows our vision for next-generation DNA storage: great potential and impressive performance are not only stored in DNA itself but also in interacting RNA and protein molecules of the eukaryotic cell, residing in the nucleus, chromosomes, and chromosomal territories. Of course, this is still a long way from technical realization. However, in the moment, there are now several spin-off ideas that are partly devoted to similar biotechnological applications.

Particularly impressive is the resilience and robustness of DNA storage in biological systems: It is passed over for generations and millions of years with only low levels of mutations and efficient proofreading. Moreover, as the biological storage system is alive, there is also error correction, repair, and even full cellular replacement of any old or dead storage units – in this sense a perfect solution from nature. For a technical device, however, using living cells or synthetic biology organisms (e.g., bacteria) as storage is not a good idea, because living cells replicate and mutate without control [27]. Hence the opposite strategy is necessary for a technical device: there are only dead components (not cells) are used and best for optimal read-in/read-out or nucleotide processing because enzymatic activity can be well controlled. For such control, light-gated protein domains that activate or block the nucleotide processing enzyme, to which they are fused to, are helpful to direct read-in and read-out of DNA storage [8]. For DNA storage devices to match the speed of modern electronic devices, it will be necessary to use further carbon-based polymers and consider their electronic properties, e.g., graphene [51] and nanocellulose [40]. This may be boosted even further by electronical signals in enzymes for substrate switching and controlling their activities [31]. Finally, nucleotides can become part of electronic components to sense and process signals even faster, for example an aptamer in a field-effect transistor [47]. Further, what we also need to implement in any high performant DNA storage device and can learn again from natural DNA and RNA storage is the inbuilt parallelism so evident in all biological systems, using millions of cells and billions of molecules in parallel. Combining these elements will help considerably and we predict that the final vision will be realized in the not-too-distant future (two decades).

## 5 Future prospects

Biology took billions of years of evolution in a continuous process of refinement of DNA storage from packing the precious DNA into histones and then nucleosomes to the rise of the eukaryotic nucleus up to full-blown chromosomes and dynamical chromosomal territories. Such a continuous process happens also regarding technical usage of DNA storage:

Already ‘simple’ bacterial plasmids show how sophisticated DNA storage can be used in molecular biology including cloning. New methods for molecular libraries and their preparation are attractive ways for DNA storage. This is used for example in the DNA–protein cross-link sequencing or Olink technology, where proteins are crosslinked or pulled down using specific antibodies in protein arrays, which are currently used in the diagnostics and analysis of important protein pathways under pathogenic conditions. Another interesting tool is to optimize individual molecules with attractive features, e.g., the polymerase or the CRISPR/Cas9 system. We should not be so conservative as to rule out there the next surprise. Thus, parallel processing of and with DNA molecules can even promote progress in the field of artificial intelligence (Ezziane, 2007).

Moreover, from the biological perspective, Table 1 and Table 2 show clearly, several types of DNA storage are already in place and nobody could think of living without them, e.g. PCR and virus diagnostics as evidenced in the corona virus pandemic. Moreover, large-scale read-out of DNA storage is a present-day reality, not only in cells, but in machines such as the Illumina sequencer. This includes latest advances such as long-read sequencing over repeat regions using PacBio approaches. However, these are the “ecological niches” where DNA storage is in place and a reality for human users. Exciting, but not easy to predict is, when DNA storage will become competitive to our best everyday large-scale storage, the electronic memory devices with storage capacities of Terabytes and more. We suspect that a break-through here for DNA storage requires synergy with electronic components, notable electronic polymers including DNA itself (e.g. DNA field effect transistors, DNA wires etc.), requiring about two decades to become real. On the other hand, development is always gradual. Thus, in human genetics technical use of DNA storage is a reality: One stores the cover slip with the individual patient DNA from Illumina sequencing in the freezer. This requires less space and cost then to buy the electronic hard drive storing the patient sequence and Illumina read-out is also cheap and fast.

**Table 2:** Synthetic biology applications of DNA storage that are inspired by nature.

**Lessons from biology**	**Optimized storage and processing applications in synthetic biology**	**Reference**
Simple polymerase	→ NGS sequencing	Jürges et al.,[38], [5], [41]
Simple biological pore	→ Nanopore sequencing, Pacific biosciences	Zhang et al.,[73]
Rolling circle replication from viruses	→ Ultra-long ssDNA chains for gene therapy	(Li et al., 2022)
Chromosomal high-density storage	→ Ultrafast sequencing cover slide as standard storage in human genetics	Rehder et al.,[52]
Random access via chromosomal territories	→ digital PCR; microtiter plates	Wöhrle et al.,[67]
Resistance factors, plasmids	→ cloning strategies	Cohen et al.,[18]
Crispr-CAS9 system	→ modern DNA editing for breeding	Gupta et al.,[30]
DNA storage in cells	→ latest DNA storage demonstrators (DNA fountains)	Erlich, Zielinski[24]
Natural computing and calculations:		
Normal brain, 1 billion neurons in parallel	→ inbuild parallelism, multi-CPU parallel processor	Du et al.,[22]

## 6 Conclusions

Nature shows how sophisticated and powerful natural DNA storage is: It allows cellular differentiation, evolution, the growth of our children and controls all our ecosystems. The components and mechanisms needed to realize DNA storage are already present in nature, whether it is the storage of data in the form of nucleic acids or the processing of such data by enzymes designed for this purpose, and our only task is to adapt them. Chromosomal territories and lncRNAs are recent high-lights on novel forms to biologically operate this storage while errors in this natural storage are important to understand pathophysiology such as cancer and natural aging. Learning from nature, we can show that in a growing number of instances some sort of DNA storage is already in technical use including PCR, genetic engineering and human genetics. Together, current DNA storage technologies allow already complete read-out of human genomes and genetic engineering of millions of base pairs. Using biology concepts from chromosomal storage, nucleic acid processing as well as polymer material sciences such as electronical effects in enzymes, graphene, nanocellulose up to DNA macramé , DNA wires and DNA-based aptamer field effect transistors will open up new applications gradually replacing classical information storage methods in ever more areas over time (decades).

## Funding

We thank Land Bavaria for support (including its contribution to DFG Project number 324392634 – TRR 221/INF) as well as the 10.13039/501100004895European Social Fund (grant ESF-ZDEX 4.0).

## CRediT (Contributor Roles Taxonomy) author statement

TD: Conceptualization, Writing - original draft. Formal analysis: AA supervised by TD. Investigation, Writing - review & editing: All authors (AA, EB, RD, TD) discussed the results and finalized the manuscript together. RD edited and polished the article including native speaker comments. Visualization: EB, AA contributed both to the design and preparation of figures. Supervision: TD.

## Author contributions

T.D. conceptualized the study and wrote the first draft. All authors wrote and edited the article. E.B. and A.A. contributed both to the design and preparation of figures. R.D. edited and polished the article including native speaker comments. All authors discussed the results and finalized the manuscript together.

## Conflict of Interest

The authors declare that the research was conducted in the absence of any commercial or financial relationships that could be construed as a potential conflict of interest.
